# Design and Validation of a Deep Learning Model for Renal Stone Detection and Segmentation on Kidney–Ureter–Bladder Images

**DOI:** 10.3390/bioengineering10080970

**Published:** 2023-08-16

**Authors:** Zih-Hao Huang, Yi-Yang Liu, Wei-Juei Wu, Ko-Wei Huang

**Affiliations:** 1 Department of Electrical Engineering, National Kaohsiung University of Science and Technology, Kaohsiung City 807618, Taiwan; f110154124@nkust.edu.tw (Z.-H.H.); i108154101@nkust.edu.tw (Y.-Y.L.); f111154140@nkust.edu.tw (W.-J.W.); 2 Department of Urology, Kaohsiung Chang Gung Memorial Hospital and Chang Gung University College of Medicine, Kaohsiung City 83301, Taiwan

**Keywords:** kidney–ureter–bladder images, renal stones, computer-aided diagnosis, deep learning, classification model, semantic segmentation

## Abstract

Kidney–ureter–bladder (KUB) imaging is used as a frontline investigation for patients with suspected renal stones. In this study, we designed a computer-aided diagnostic system for KUB imaging to assist clinicians in accurately diagnosing urinary tract stones. The image dataset used for training and testing the model comprised 485 images provided by Kaohsiung Chang Gung Memorial Hospital. The proposed system was divided into two subsystems, 1 and 2. Subsystem 1 used Inception-ResNetV2 to train a deep learning model on preprocessed KUB images to verify the improvement in diagnostic accuracy with image preprocessing. Subsystem 2 trained an image segmentation model using the ResNet hybrid, U-net, to accurately identify the contours of renal stones. The performance was evaluated using a confusion matrix for the classification model. We conclude that the model can assist clinicians in accurately diagnosing renal stones via KUB imaging. Therefore, the proposed system can assist doctors in diagnosis, reduce patients’ waiting time for CT scans, and minimize the radiation dose absorbed by the body.

## 1. Introduction

Current research indicates a global increase in the incidence of renal stones, as observed in various studies conducted worldwide (including Italy, Germany, Scotland, Spain, Sweden, Japan, and the United States) [1,2,3]. Several diagnostic and treatment approaches have been proposed to address the growing prevalence of renal stones.

**Figure 1:**
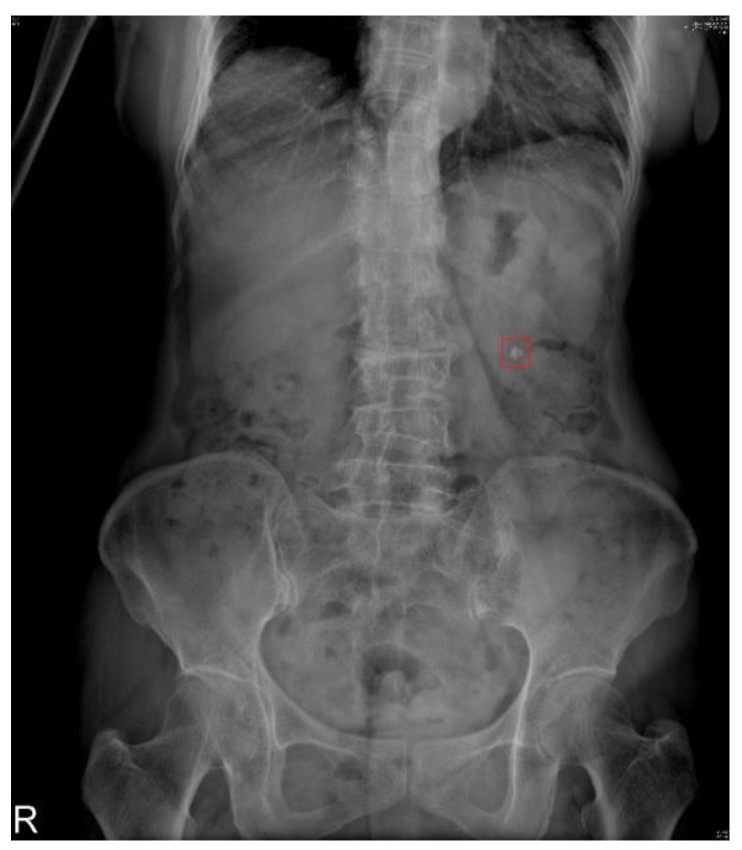
Urinary tract stones on a kidney–ureter–bladder (KUB) image and red box mean the location of the stone.

X-rays are low-cost and low-radiation imaging modalities that are widely used in various stutabledies for computer-aided diagnosis (CAD) development, including chest X-rays for COVID-19 detection with DL models [4], breast X-rays for detecting breast cancer [5], and abdominal X-rays for assisting in the diagnosis of muscle coordination disorders [6]. In this study, X-ray kidney–ureter–bladder (KUB) images were used (Figure 1). In this figure, the white area inside the red frame is a urinary tract stone, as a specialist would diagnose. KUB imaging has a few limitations, including its two-dimensional nature, which may lead to false positives and make it difficult to distinguish between abnormalities in high-density tissues [7]. The sensitivity of KUB imaging in detecting renal stones ranges from 44 to 77%, with a specificity of 80–87% [8], whereas computed tomography (CT) has a sensitivity of 94–100% and a specificity of 92–94.2% [9,10]. Noncontrast CT is the most accurate imaging modality for kidney stones owing to high sensitivity, specificity, accurate stone sizing, and the ability to evaluate non-stone-related pathologies [11].

CT is commonly used for whole-body screening to detect tumors or inflammation in organs and is highly reliable for diagnosing diseases such as liver, lung, and brain cancers [9,10]. Several studies have focused on various CT examinations, including deep learning (DL)-based detection of hemorrhagic lesions on brain CT images and segmentation [12], and distinguishing COVID-19 severity by analyzing the lung opacity on chest CT images [13]. Abdominal CT exhibits higher sensitivity than conventional radiography in detecting calcifications and promptly diagnosing urinary tract stones, while also being able to detect uric acid or cystine stones unaffected by obstruction [14]. Furthermore, CT imaging can assist clinicians in accurately diagnosing patients with symptoms arising from factors such as inflammation in the abdominal cavity, vascular abnormalities, or urinary system tumors [4]. CT imaging provides a three-dimensional (3D) visualization of the urinary system, including the kidneys, ureters, and bladder, enabling clinicians to promptly understand the patient’s condition. In most cases, noncontrast computerized tomography (CT) is recommended for diagnosing ureteral stones; a low-energy protocol is suggested if the patient’s body habitus is favorable. Conventional radiography and ultrasound are utilized to monitor the passage of the majority of radiopaque stones, as well as for most patients undergoing stone removal. [15]

However, CT is more expensive and produces higher radiation doses than X-ray imaging. For adult abdominal X-ray examinations, the radiation dose of CT in the same area ranges from 8 to 34 mGy [16,17], whereas that of X-rays is approximately 2.47 mGy [18]. Additionally, in other areas, such as the stomach, the radiation dose of CT is approximately 50 times higher than that of X-rays [19]. These factors pose a risk to human health. In recent years, several clinical techniques were developed to address these issues, including low-dose CT, which reduces the radiation dose produced during a routine CT scan. For example, the radiation dose of abdominal CT scans has been reduced from 25 to 17 mGy. However, even with low-dose CT, the radiation dose is still approximately seven times higher than that of X-rays [20]. Considering these challenges, X-ray imaging has emerged as a low-cost and low-radiation examination method with minimal impact on human health. Nevertheless, compared with CT, X-rays have lower sensitivity and are less effective in detecting smaller renal stones. Therefore, improving the sensitivity of radiography for diagnosing renal stones could lead to wider application and effectively reduce medical costs.

Recently, the rapid development of machine learning and artificial intelligence has facilitated the application of CAD in medical image processing. The exceptional performance of convolutional neural networks (CNNs) in learning and computation across various domains [21,22,23,24] has led to their widespread adoption in CAD. The accuracy of CAD models incorporating CNNs has gradually improved over time [25,26]. Recent studies have applied neural networks to diagnose urinary system diseases using CT imaging [27,28,29]. CNN models have exhibited a sensitivity of 89.6% and a positive predictive value of 56.9% in detecting urinary stones during X-ray examinations [30]. Liu et al. [31] combined image preprocessing and data augmentation techniques with the ResNet model to detect renal stones on KUB images, and achieved an accuracy, sensitivity, specificity, and F1-measure of 0.982, 0.964, 1.000, and 0.982, respectively.

KUB imaging remains the primary examination method for detecting urinary stones in emergency rooms owing to its convenience, affordability, and low radiation dose. However, only experienced urologists and radiologists can accurately diagnose urinary stones from KUB images. Inexperienced physicians may make errors or further prescribe CT scanning, thus increasing the medical costs and radiation exposure. Compared to deep learning, traditional image processing algorithms do not demonstrate robust generalization capabilities, mainly because of the large size of KUB images and the small dimensions of kidney stones. Furthermore, conventional approaches face challenges when effectively detecting irregular features. To address this issue, we developed a CAD system based on a DL model to assist emergency physicians in accurately diagnosing urinary stones based on KUB imaging. The system was validated through experimental data and specialist evaluations.

## 2. Materials and Methods

### 2.1. Molecular Structure of Renal Stones

Renal stones consist of urinary solutes (such as calcium oxalate and uric acid) in an unstable supersaturation state (including hypercalciuria, hyperoxaluria, and hyperuricosuria) due to imbalances between the promoters and inhibitors of stone formation. Renal stone formation occurs in four phases: nucleation, crystal growth, aggregation, and retention [32]. Finally, these stones remain in the collecting system of the kidneys and migrate to the urinary tract. Larger renal stones that cannot pass through the body may cause various health problems, including hematuria, renal colic pain, urinary tract infection, hydronephrosis, and renal function impairment.

The primary classifications of renal stones with their molecular formula and occurrence are listed as follows [32]:

- Calcium (Ca)-containing stones:

Calcium oxalate, CaC_2_O_4_ (H_2_O)_2_ or CaC_2_O_4_H_2_O (60%)

Hydroxyapatite, Ca_10_(PO_4_)_6_(OH)_2_ (20%)

Brushite, CaHPO_4_·(H_2_O)_2_ (2%)

- Non-calcium-containing stones:

Uric acid, C_5_H_4_N_4_O_3_ (7%)

Struvite, NH_4_MgPO_4_·(H_2_O)_6_ (7%)

Cystine, C_6_H_12_N_2_O_4_S_2_ (1–3%)

Ca-containing stones represent the majority of renal stones, making it theoretically possible to detect most renal stones using X-ray imaging. KUB imaging, a rapid and cost-effective X-ray imaging technique, is an ideal first-line examination for renal stone detection.

### 2.2. Datasets

**Figure 2:**
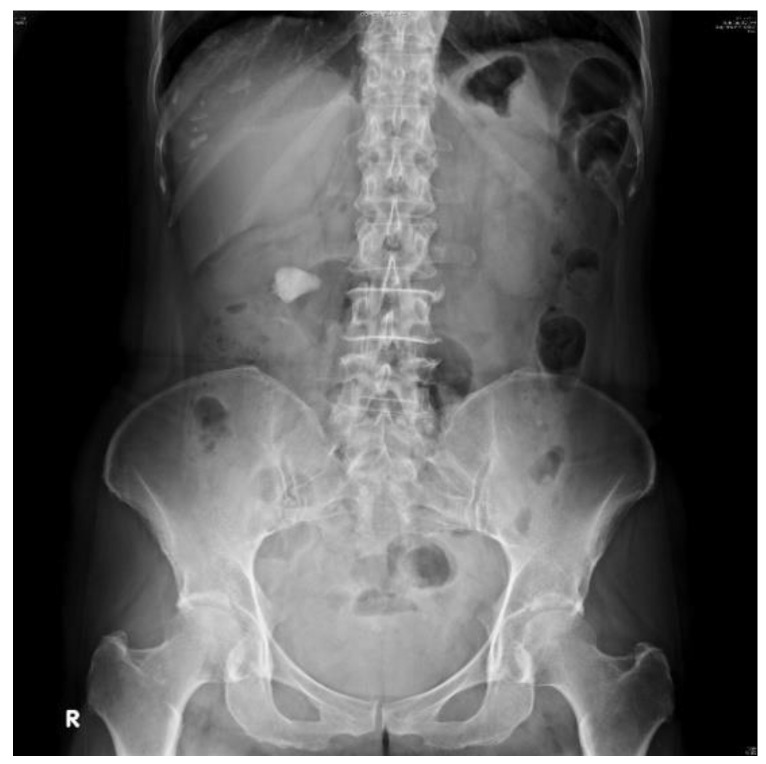
KUB image showing urinary stones.

We collected 485 abdominal radiographs of patients diagnosed with upper urinary tract stones by urologists at Kaohsiung Chang Gung Memorial Hospital. Owing to the varying lengths of the follow-up, each patient had KUB images taken at different times, and some KUB images contained multiple urinary tract stones; therefore, they were divided into single or multiple training images with urinary tract stones (Figure 2).

### 2.3. Image Preprocessing

#### 2.3.1. Contrast-Limited Adaptive Histogram Equalization

Histogram equalization (HE) [33] enhances image contrast and suppresses noise. Adaptive histogram equalization (AHE) [34] further enhances local contrast by dividing the image into multiple regions and applying HE to each region. However, AHE can cause image distortion due to excessive enhancement of local contrast and does not address noise amplification in dark areas. Contrast-limited AHE (CLAHE) [35] avoids discontinuities and excessive local contrast caused by AHE by controlling the slope of the cumulative density function (CDF). An abrupt increase in the slope of the CDF indicates a high grey value in the region, whereas a decrease indicates a low grey value. CLAHE limits gray values that exceed a certain threshold and redistributes them to various gray levels, resulting in a smoother CDF that can be efficiently calculated using linear interpolation. This method effectively suppresses noise and enhances the contrast between the urinary stone and the background, making it particularly useful for images with very dark or bright backgrounds. CLAHE is widely used in medical imaging [36,37,38].

**Figure 3:**
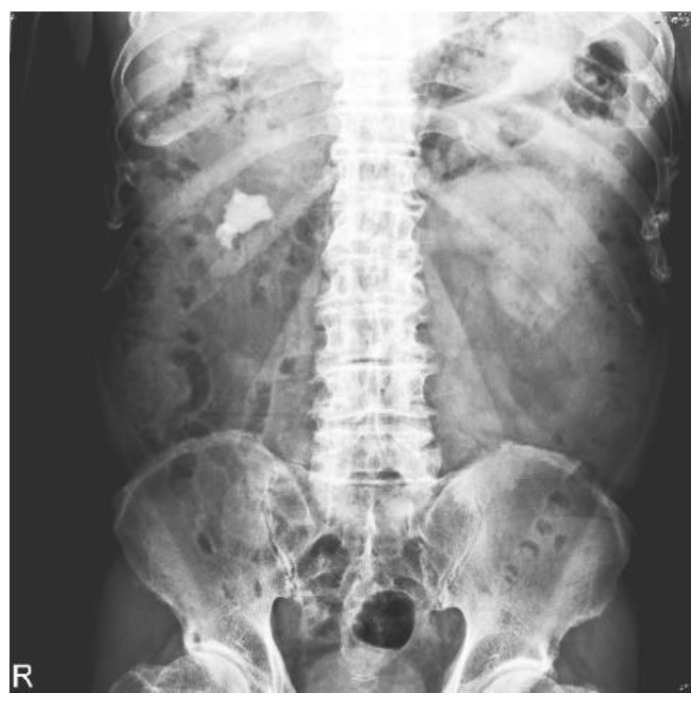
KUB image after histogram equalization.

**Figure 4:**
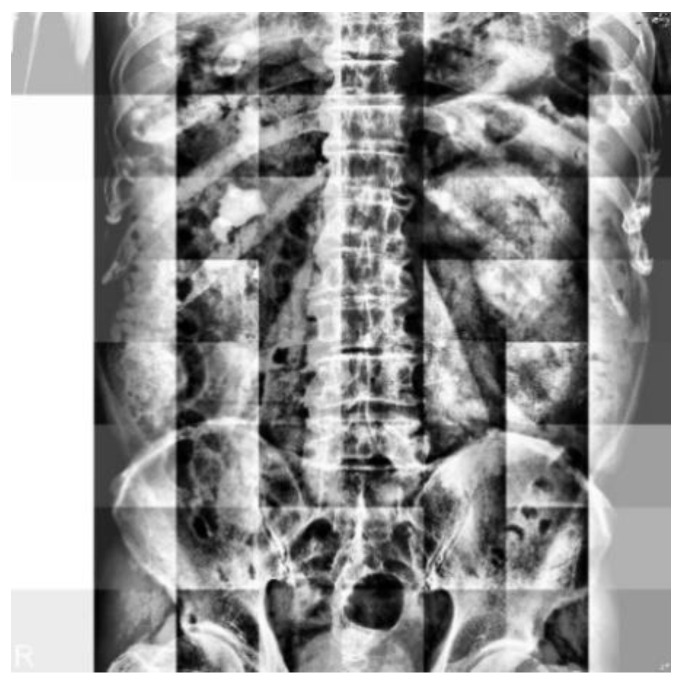
KUB image after AHE with an 8 × 8 mask.

**Figure 5:**
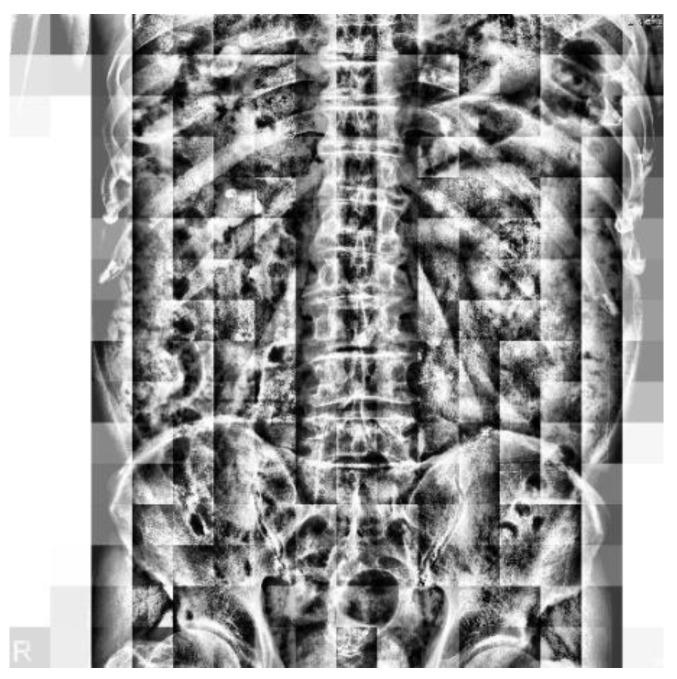
KUB image after AHE with a 16 × 16 mask.

**Figure 6:**
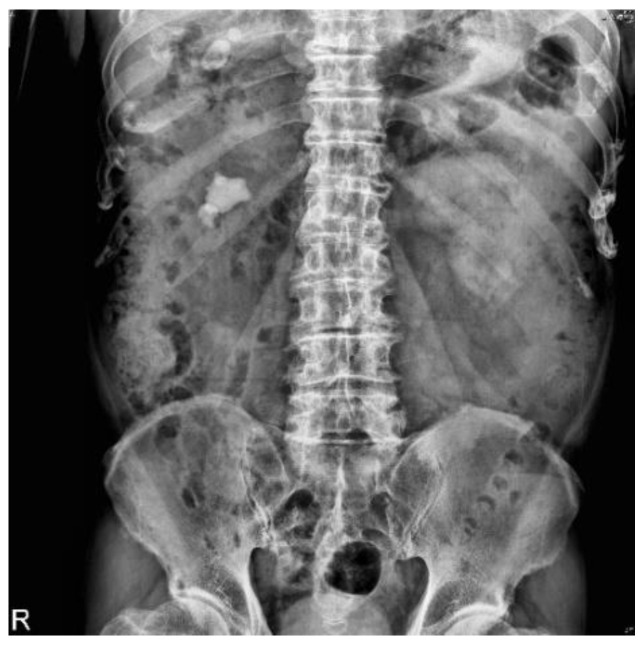
KUB image after contrast-limited AHE.

Figure 3 illustrates several areas in the histogram-equalized image that are already overexposed, particularly high-density areas such as bones, which are prominent. Figure 4 shows AHE with an 8 × 8 mask, which increases the local details; however, inconsistency between the blocks causes pixel discontinuity in the entire image. Figure 5 depicts AHE with a 16 × 16 mask, where the pixel discontinuity is even more obvious. Contrast-limited AHE reduces overexposure in the histogram-equalized image and does not cause pixel discontinuity, resulting in a square-like appearance of the image and enabling observation of urinary stones on the image (Figure 6). Therefore, we applied this method to KUB images in this study.

#### 2.3.2. Image Mask

**Figure 7:**
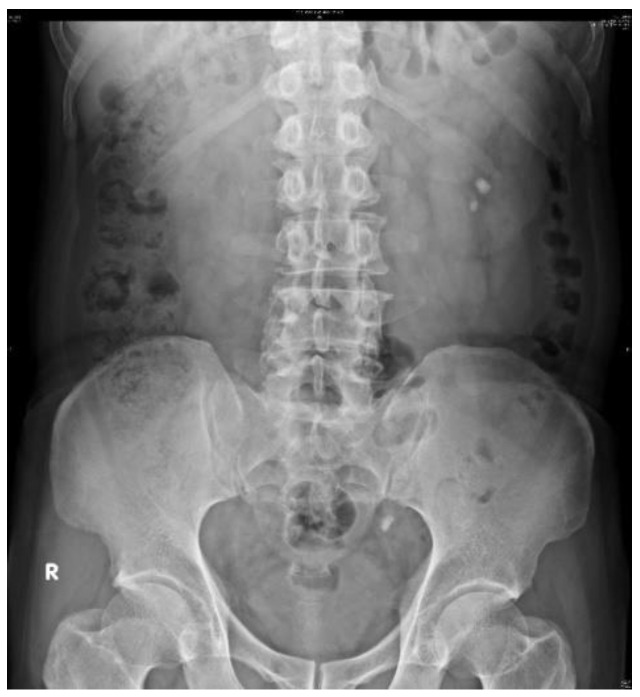
Original KUB image.

**Figure 8:**
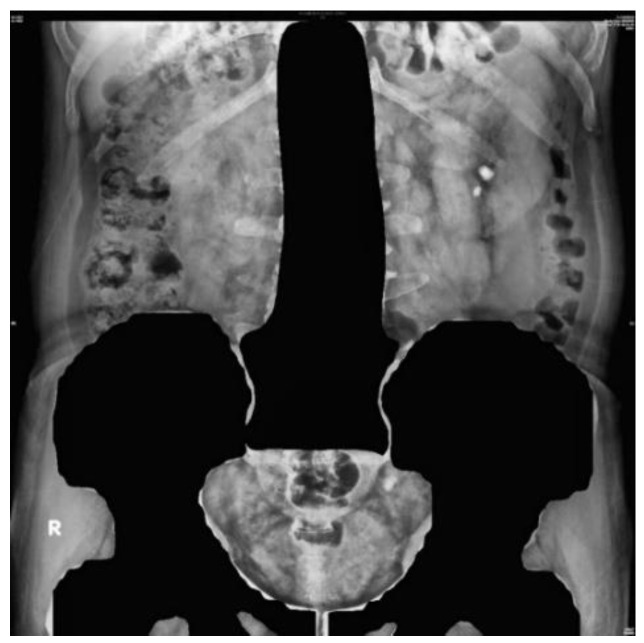
Masked image generated by Mask R-CNN.

**Figure 9:**
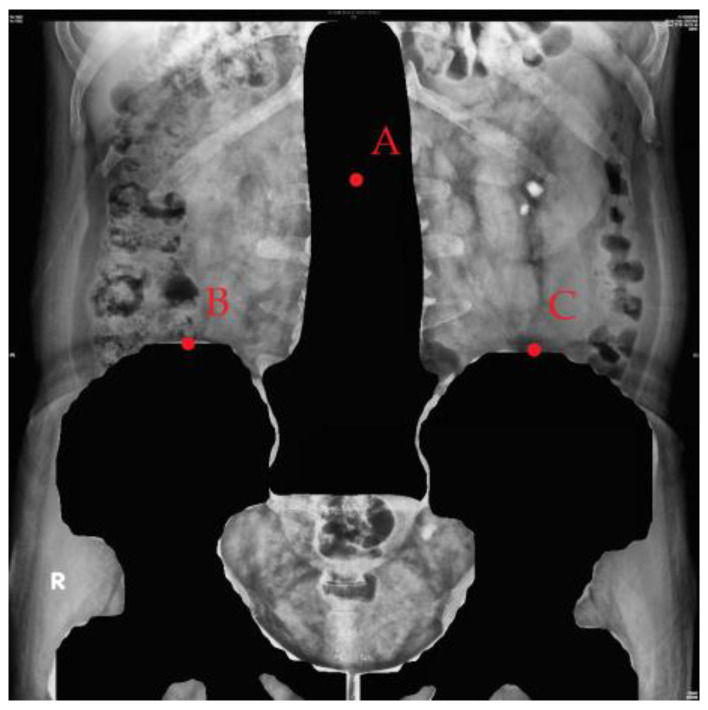
A is the central point of spine, B is the upper point of the right pelvis, and C is the upper point of the left pelvis.

**Figure 10:**
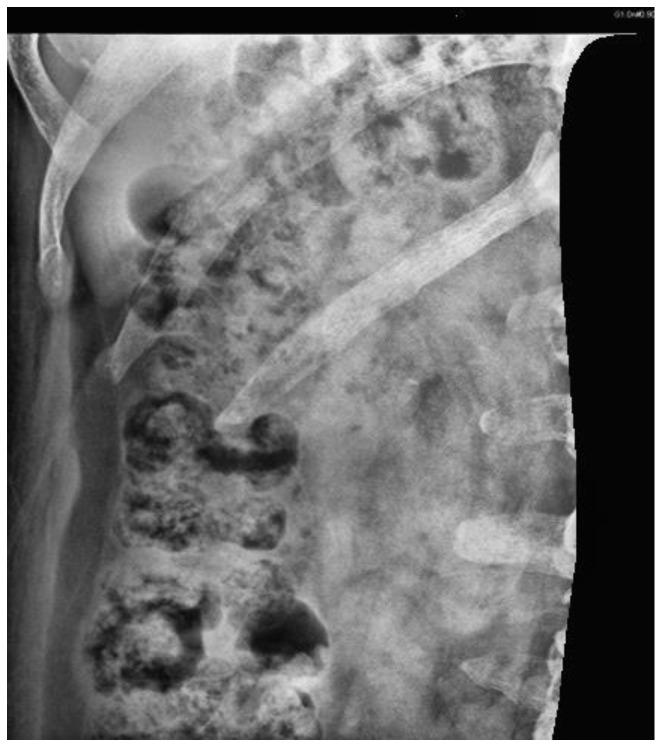
KUB image of the right kidney.

**Figure 11:**
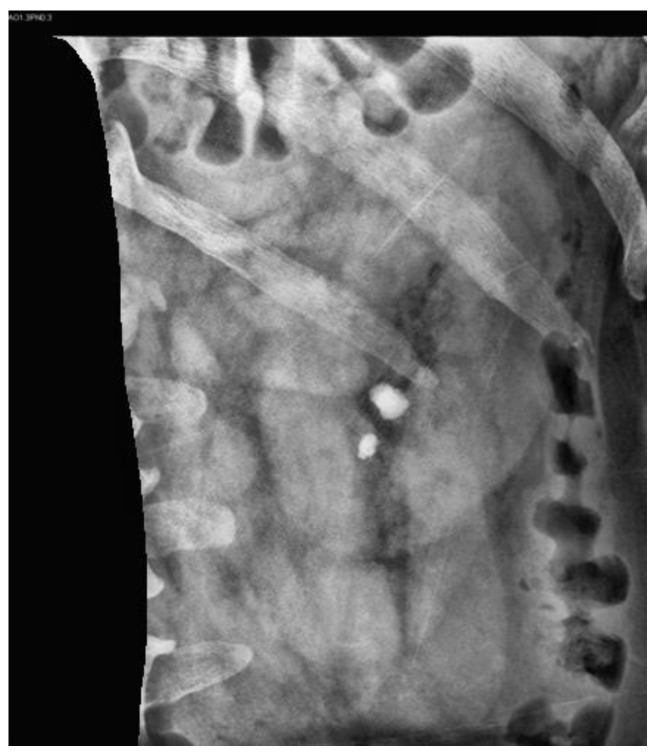
KUB image of the left kidney.

Figure 7 illustrates the original KUB image. First, all KUB images were processed using an image segmentation network model, Mask R-CNN [39], which was trained to detect the spine and pelvis [40] to generate masks that block most of the bright areas in the KUB images (Figure 8). The images were then segmented about the central point of the spine and upper pelvis on both sides (Figure 9, Figure 10 and Figure 11), respectively. Masks were used for segmentation because abnormalities are difficult to detect in high-density tissues on X-ray images; the higher the density of the tissue, the brighter it appears on the image [7].

#### 2.3.3. Image Cropping

**Figure 12:**
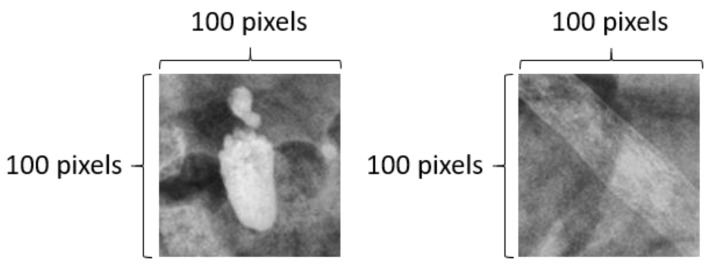
Left-hand-side image shows the stone image cropped from the KUB image with a size of 100 × 100 pixels, while the right-hand-side image shows the randomly cropped image with the same size from the KUB image.

A 100 × 100-pixel image was cropped from the KUB image. An image with urinary tract stones was positioned at the center of the image. Cropped images without urinary tract stones were randomly selected from the KUB images, as illustrated in Figure 12. Based on a 100 × 100-pixel image, we introduced the concept of a sliding window for real-world applications. A sliding window is used to capture the presence of stones in the image. If stones were detected in the cropped image, we would map them back to the original KUB image and obtained their location. Once the full-image scan was complete, we extracted the image based on these specific locations and performed mask detection using the segmentation model. After mask detection was complete, we merged it back with the original image via mapping and positioning, thereby obtaining a complete KUB stone mask image.

### 2.4. Data Augmentation

Studies have shown that data augmentation can effectively prevent overfitting of the model, with the probability of overfitting in small datasets during training being higher than in large datasets [41,42,43,44]. However, there are multiple fields in which large amounts of data are not available for research, such as medical image analysis. Studies on medical image analysis have used more than 4000–5000 images for training [45,46,47,48]; however, in the field of DL, this is still considered a small dataset. Therefore, data augmentation can be used to increase the variation in images, which not only avoids the problem of low accuracy due to insufficient data but also increases the diversity of samples and improves the generalization ability of the model [49,50,51].

**Figure 13:**
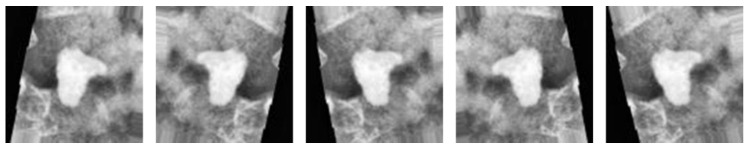
After cropping the KUB image, random angle rotation, horizontal and vertical displacements, and flipping are applied to augment data.

To increase the amount and diversity of data, random angle rotation, horizontal and vertical displacements, and flipping were applied to the original images (Figure 13) [52]. In this study, random data augmentation was applied to the training dataset during each iteration. When the augmented images were generated first and then used for model training, all data were written into the memory before training. However, by dynamically generating augmented image data during the iteration, only the original image data were read, which reduced memory consumption.

### 2.5. Deep Learning Model

#### 2.5.1. Residual Network

**Figure 14:**
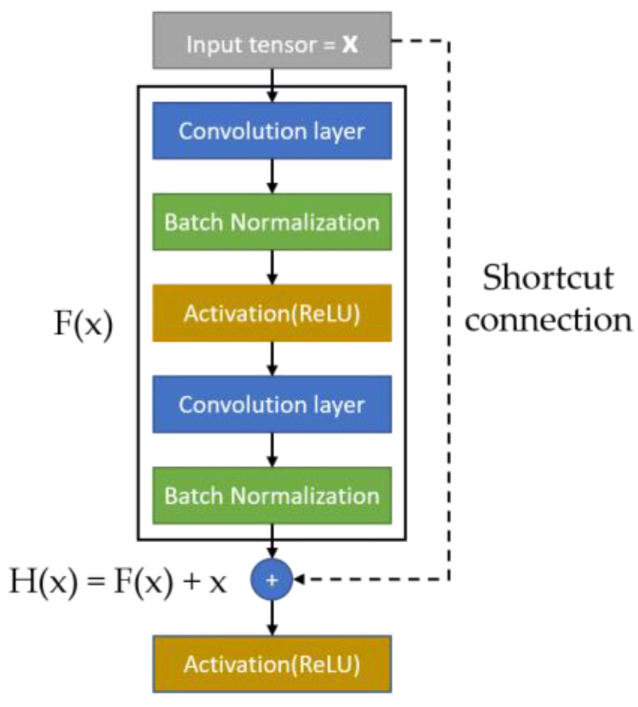
Residual block [52].

Previous studies have shown that the deeper the CNN, the finer the features it can extract [53]. However, in 2016, Kaiming discovered that the model’s performance decreased and experienced degradation when the network layer became excessively deep [54]. To solve this problem, they proposed a network structure called ResNet, which introduced the concept of a residual block. The residual block copies the output of the source layer directly to the shortcut connection and adds it to the output of the main framework, as illustrated in Figure 14. If the layers in the residual block do not learn any features, the output of the block is the same as the input; this is called identity mapping. Residual blocks address the problem of model degradation caused by overly deep networks, allowing the number of network layers to increase.

#### 2.5.2. Inception-ResNetV2

**Figure 15:**
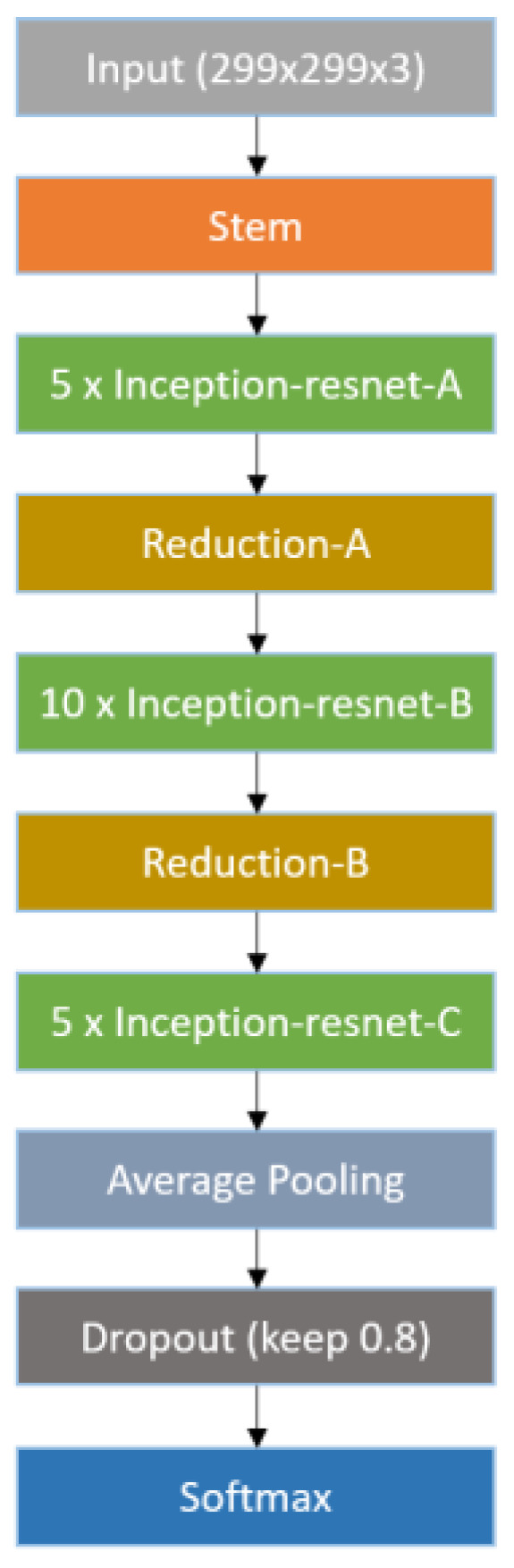
Main structure of Inception-ResNetV2 [55].

In 2016, Google proposed Inception-ResNetV2 as an improved version of Inception-ResNetV1 [55], which achieved the best performance in the ILSVRC image classification benchmark test [56]. The core concept of Inception-ResNetV2 is to combine inception modules and residual direct connections using residual connection shortcuts to successfully train deeper neural networks while significantly simplifying inception modules. As indicated in Figure 15, the structure of Inception-ResNetV2 is divided into several parts: Stem, Inception-Resnet-A, Reduction-A, Inception-Resnet-B, Reduction-B, and Inception-Resnet-C. The A, B, and C modules use asymmetric convolutional layers and 1 × 1 convolutional layers to reduce or unify dimensions, and modules A and B are designed to gradually reduce the size of the feature map to avoid the loss of related information. By combining the above modules, Inception-ResNetV2 can achieve a deep network architecture without encountering the problems of gradient disappearance and can converge better. Recently, Inception-ResNetV2 has been increasingly used for medical image recognition. For example, a previous study [57] explored the application of Inception-ResNetV2 for brain tumor detection. Other studies have also implemented Inception-ResNetV2 in various applications, such as skin lesion classification methods [58,59] and benchmark testing for aortic pathology analysis [60].

#### 2.5.3. U-Net

**Figure 16:**
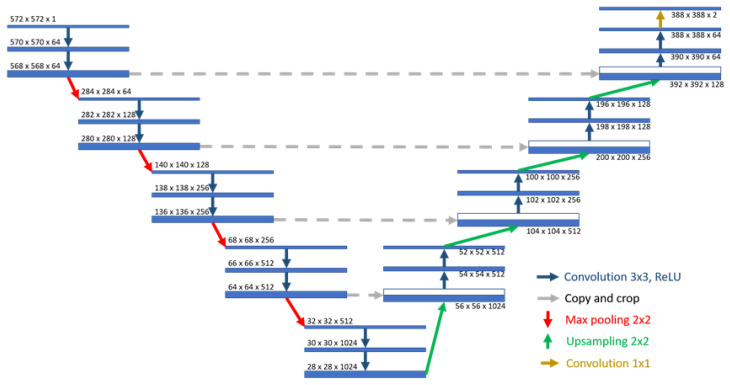
U-Net architecture [61].

Proposed in 2015, U-Net is widely used in medical image segmentation owing to its unique structure [61] (Figure 16). The structure of U-Net can be conceptualized as an encoder–decoder structure. The encoder comprises four sub-modules, each containing two convolutional layers, followed by a max pooling layer for downsampling. These modules gradually decrease the resolution of the image. The decoder is comprised of four sub-modules that progressively upsample the image resolution until it matches the input image resolution. U-Net also adopts the technique of skip connections, which connects the upsampled results of the decoder with the outputs of the encoder submodules of the same resolution as the input to the next submodule. The feature concatenation is unique as it concatenates the features in the channel dimension to form thicker features, thus avoiding information loss during feature propagation. Several improved versions based on U-Net are available, such as 3D U-Net [62], which is used for the segmentation of 3D images; Res-UNet [63], which combines the concept of ResNet using residual blocks instead of convolutional layers; and ResUnet++ [64], which introduces attention modules [65] and ASPP modules [66].

### 2.6. System Architecture

**Figure 17:**
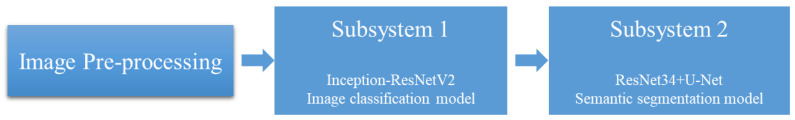
Schematic of the overall system architecture.

**Figure 18:**
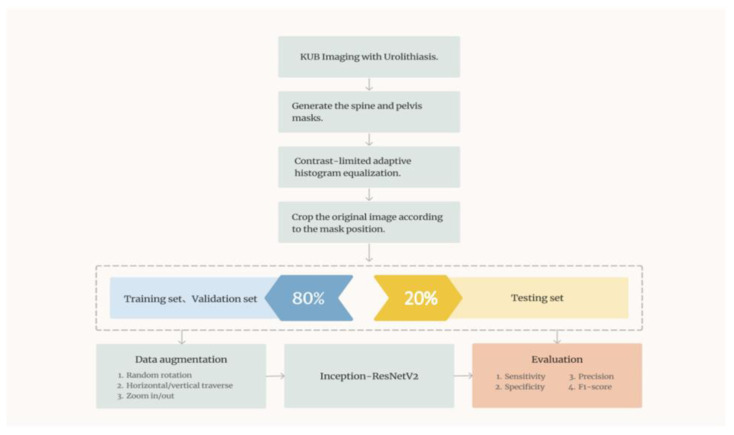
Architecture of the image classification model.

**Figure 19:**
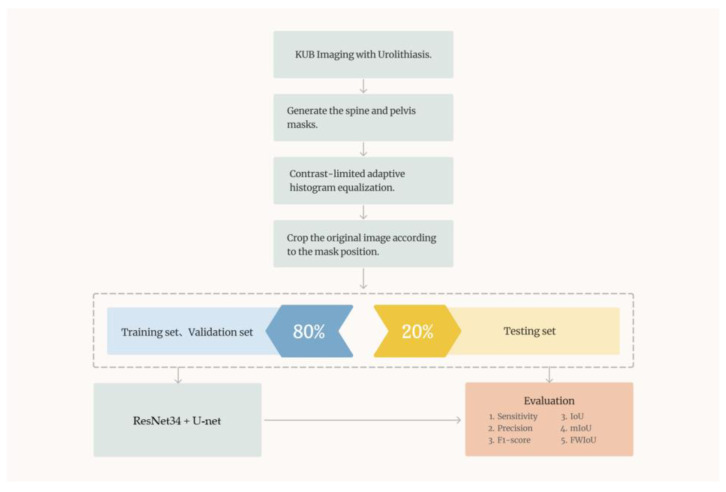
Architecture of the semantic segmentation model.

**Figure 20:**
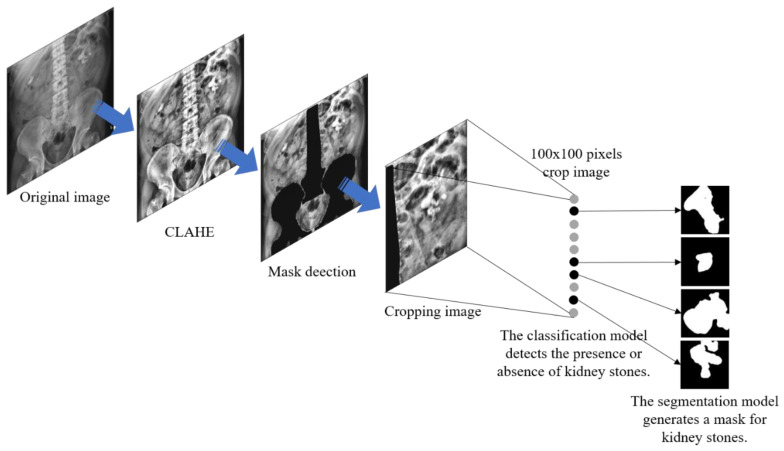
Complete system flow for visualizing KUB images.

**Figure 21:**
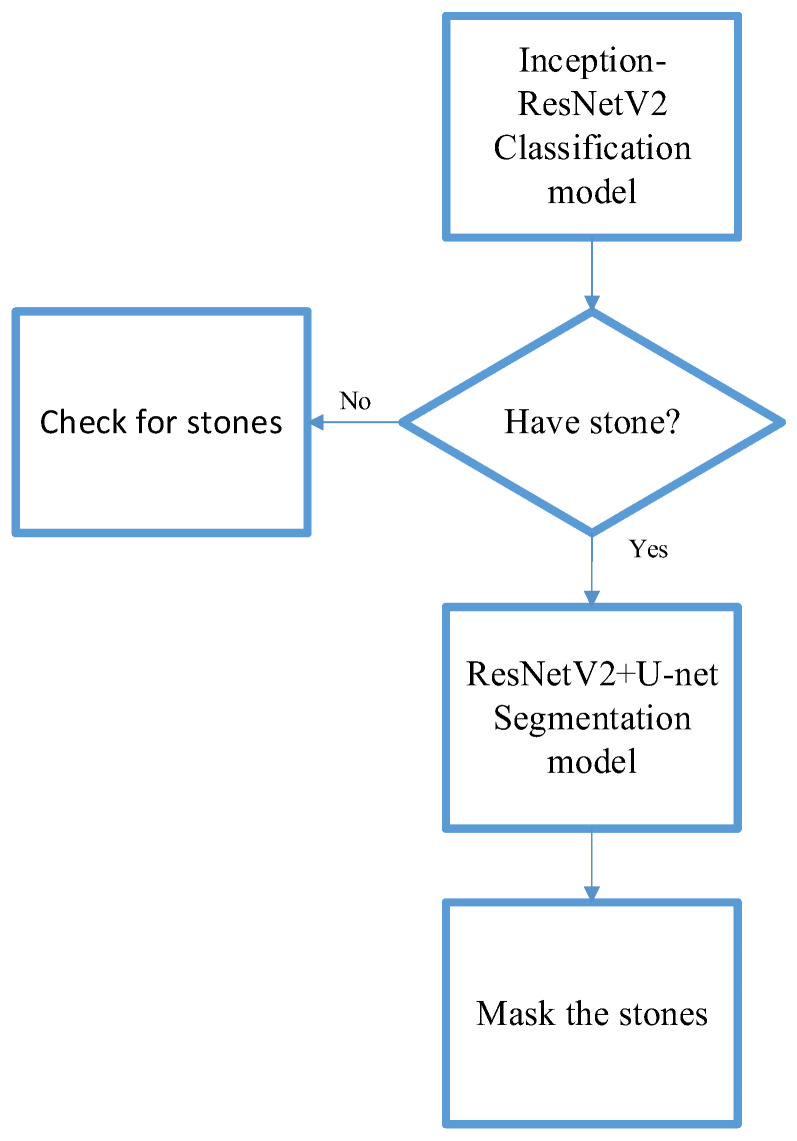
System flow chart.

In this study, we proposed a computer-aided diagnostic system consisting of two subsystems. The overall system architecture is illustrated in Figure 17. Subsystem 1 is a urinary stone classification model based on Inception-ResNetV2, which is shown in Figure 18. Subsystem 2 is a urinary-stone segmentation model based on U-Net, which is illustrated in Figure 19. The system first generated a mask to remove the spine and pelvis from the KUB images and then performed limited-contrast AHE on the images. The kidney area was approximately segmented according to the mask, and 100 × 100-pixel stone images were cropped. The dataset was divided into training and testing sets in the ratio of 8:2, and data augmentation was employed by the classification model to simulate the diversity of stone images. After training the Inception-ResNetV2 classification model, the system was evaluated using several metrics, including sensitivity, specificity, precision, and F1-measure. The architecture of the semantic segmentation model was similar to that of the classification model, except for the data augmentation component and inclusion of three additional evaluation metrics for the mask: IoU, MIoU, and FWIoU. The complete system flow for visualizing KUB images is shown in Figure 20. The flowchart of the computer-aided diagnostic system is depicted in Figure 21.

## 3. Results

Windows 10 was used as the operating system for testing the model; the hardware information is listed in Table 1. A Python 3.7 environment on Anaconda 3 with a Tensorflow-GPU version was used to train the neural network, which was built and trained using Keras.

### 3.1. Evaluation Metrics

In this study, all images were divided into three datasets, with a total of 1340 images. Among these, 970 images were used for training (80%) and validating (20%) the model; the training set contained 776 images, and the validation set 194 images. The remaining 370 images were used as the test set to evaluate the performance of the model and its generalization ability. Both subsystems used Ranger as the optimizer [67], which is an integration of two optimizers: RAdam [68] and LookAhead [69]. The loss function used in subsystem 1 was binary cross-entropy. For the semantic segmentation model, ResNet50 was used as the primary feature extractor network, and U-net utilized the features for prediction and mask generation. Ranger was used as the optimizer, and the loss function was composed of binary cross-entropy and Jaccard distance.

**Figure 22:**
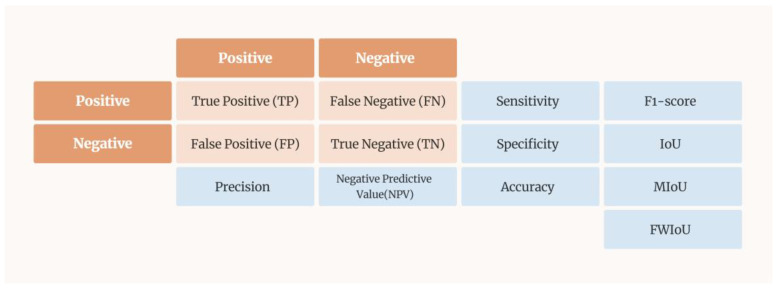
Confusion matrix and evaluation metrics.

We generated a confusion matrix from the prediction results, which had four categories of correct and incorrect predictions. The categories for correct predictions were true positive (TP) and true negative (TN), whereas those for incorrect predictions were FP and FN. The confusion matrix is depicted in Figure 22. We used these four categories to generate seven metrics for evaluating the performance of the model. The formula for the accuracy is as follows:

(1) $$Accuracy=\frac{TP+TN}{TP+TN+FP+FN}$$

In addition to determining the model’s accuracy, we used seven other metrics, four of which were used to evaluate the classification and semantic segmentation models: sensitivity, specificity, precision, and F1-measure. The other three metrics, IoU, MIoU, and FWIoU, were used to evaluate the quality of the predicted masks of the semantic segmentation model. The formula for sensitivity is as follows:

(2) $$Sensitivity=\frac{TP}{TP+FN}$$

The formula for specificity is as follows:

(3) $$Specificity=\frac{TN}{FP+TN}$$

The formula for precision is as follows:

(4) $$Precision=\frac{TP}{TP+FP}$$

We used the F-measure to comprehensively evaluate the performance of the model. The higher the F1-measure value, the better the performance of the model. The formula for the F1-measure is as follows:

(5) $$F_{\beta }–measure=(1+\beta^{2})\frac{Precision\times Recall}{(\beta^{2}\times Precision)+Recall}$$

The formula for the IoU is as follows:

(6) $$IoU=\frac{TP}{TP+FP+FN}$$

The formula for the MIoU is as follows:

(7) $$MIoU=(\frac{TP}{TP+FP+FN}+\frac{TN}{TN+FN+FP})/2$$

The FWIoU is a modification of the MIoU in which weights are assigned based on the frequency of occurrence of each class. The formula for the FWIoU is as follows:

(8) $$FWIoU=\left(\frac{TP+FN}{TP+FP+TN+FN}\right)\times \frac{TP}{TP+FP+FN}$$

### 3.2. Effect of Data Augmentation on the Training of the Classification Model

**Figure 23:**
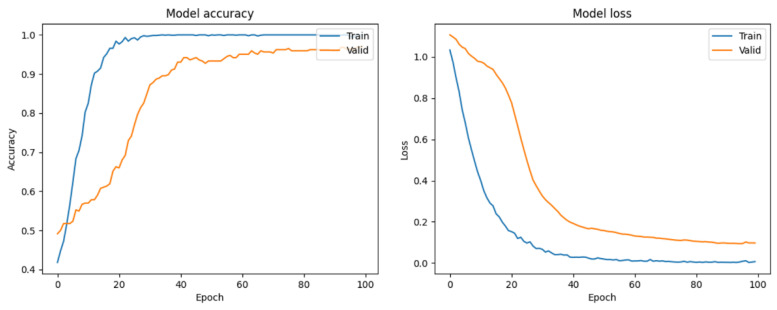
Accuracy and loss of the ResNet50 model without data augmentation.

**Figure 24:**
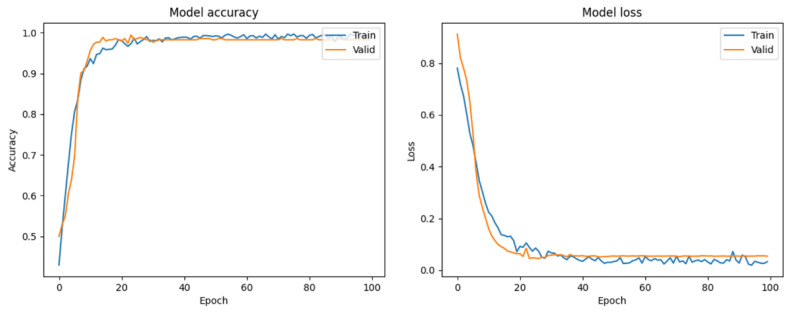
Accuracy and loss of the ResNet50 model with data augmentation.

In this study, ResNet50 models were trained using both augmented and nonaugmented datasets. Data augmentation was performed by rotating, horizontally and vertically shifting, and magnifying and demagnifying of the original images. The difference between the effects of using and not using data augmentation was compared based on the accuracy and loss during the training process of the ResNet50 model. Figure 23 shows the updates of accuracy and loss during the training process of the model without data augmentation. The accuracy of the model without data augmentation improved faster during training than that during validation. In contrast, Figure 24 shows the updates of accuracy and loss during the training process of the model with data augmentation. The accuracies of the training and validation datasets were similar. The X-axes on the left-hand sides of Figure 22 and Figure 23 represent the training steps, whereas the Y-axes represent the accuracy. In Step 10, the accuracy of the training dataset in Figure 23 is approximately 0.9, but the accuracy of the validation dataset is only approximately 0.55. However, in the same step shown in Figure 24, the accuracy of the training dataset is approximately 0.9, and the accuracy of the validation dataset is also improved to approximately 0.9. We observed that data augmentation resulted in a certain degree of improvement in the training and generalization ability of the model.

### 3.3. Subsystem 1—Classification Model for Medical Images

**Figure 25:**
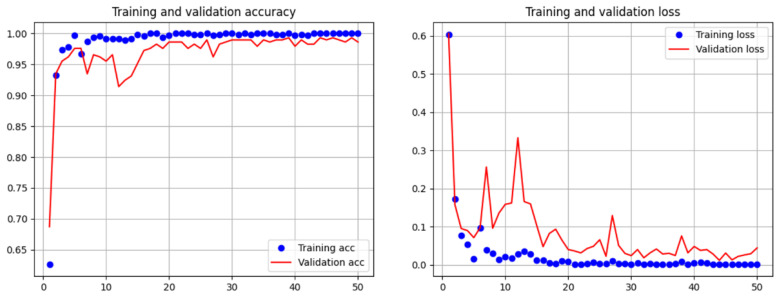
Accuracy and loss updates during the training process of ResNet50.

**Figure 26:**
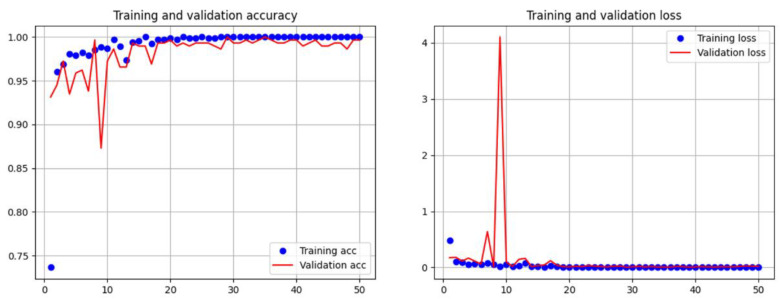
Accuracy and loss updates during the training process of Inception-ResNetV2.

In this study, we trained two models, ResNet50 and Inception-ResNetV2, for 50 epochs with an initial learning rate of 0.001. The specific model initialization parameters are listed in Table 2. An excessively small learning rate could slow the convergence and increase the training time, whereas an excessively large one can cause parametric oscillations. Therefore, choosing an appropriate initial learning rate and appropriately reducing it after multiple epochs can improve the model’s performance. If the validation loss function did not continue to decrease after five consecutive epochs, the learning rate was multiplied by 0.5. Figure 25 illustrates the accuracy and loss updates during training. Table 3 lists the confusion matrix of the model’s predictions on the test set, and Table 4 shows the calculated accuracy, sensitivity, specificity, precision, and F1-measure based on the confusion matrix, which were 0.989, 0.995, 0.984, 0.984, and 0.989, respectively. The Inception-ResNetV2 model used the same parameter settings as those of ResNet50. Figure 26 depicts the accuracy and loss updates during training. The confusion matrix of the test set is shown in Table 5. As summarized in Table 6, the accuracy, sensitivity, specificity, precision, and F1-measure calculated based on the confusion matrix were 0.997, 1.000, 0.995, 0.995, and 0.997, respectively. Table 7 presents the comparison of the test results of ResNet50 [31] with those of Inception-ResNetV2. The results of all indicators were higher for Inception-ResNetV2.

### 3.4. Subsystem 2—Segmentation Model for Medical Images

**Figure 27:**
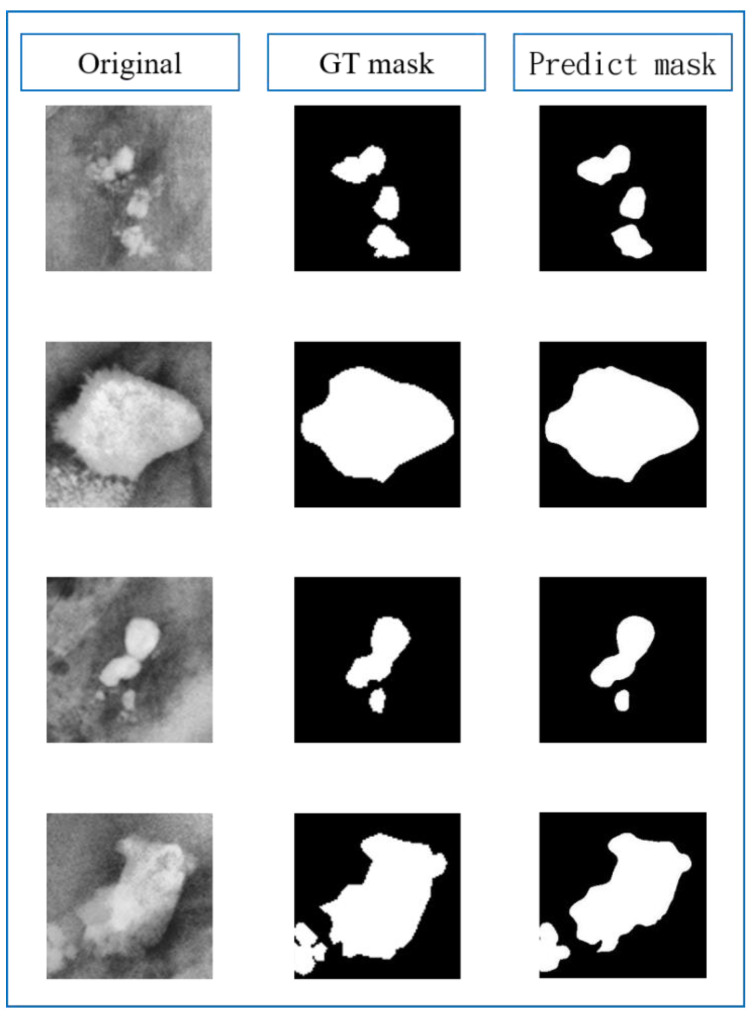
Original image, ground truth mask, and predicted mask.

This study employed two different backbone networks, ResNet34 and ResNet50, to implement four U-net models using different loss functions, including bce_dice_loss, bce_jaccard_loss, binary_focal_dice_loss, and binary_focal_jaccard_loss, as shown in Equations (9)–(12), respectively. The specific U-net model initialization parameters are listed in Table 8. Table 9 and Table 10 show the confusion matrices for each model and loss function. According to Table 9, a higher false negative (FN) value indicates that the model failed to detect a portion of the actual mask, resulting in a larger area of the actual stone being missed. A false positive (FP) value indicates misjudgment by the model, resulting in a mask area that does not contain stones. As the segmentation model in this study primarily divides the image into foreground (urinary stone image or positive) and background (negative), the evaluation scores were calculated separately for the foreground and background based on the confusion matrix, with the scores presented in Table 11, Table 12 and Table 13. Table 9 and Table 10 reveal a vast difference in the number of samples between the foreground and background. Focal loss is primarily introduced as a loss function to resolve the imbalance between positive and negative samples. Therefore, Table 11 and Table 12 indicate a subtle improvement in the model’s performance when using focal loss compared to binary cross-entropy. Based on the evaluation metrics, both ResNet34 and ResNet50 effectively predict the urinary stone masks. The frequency-weighted intersection over union (FWIoU) index, which assigns different IoU weights to each label based on the test set data, is a valuable indicator for comprehensively evaluating the model’s performance. Therefore, based on the mean IoU (MIoU) and FWIoU, the best performance was achieved by combining ResNet34 as the backbone network with U-net and using binary cross-entropy plus Jaccard distance as the loss function, with sensitivity, precision, F1-score, IoU, MIoU, and FWIoU of 0.952, 0.984, 0.968, 0.937, 0.834, and 0.905, respectively. Figure 27 illustrates the original image, ground truth mask, and predicted mask.

(9) $$bce_{dice_{loss}}=\left(-\frac{1}{N}\sum_{i=1}^{n}y_{i}\cdot log\hat{y_{i}}+(1-y_{i})\cdot log(1-\hat{y_{i}})\right)+\;(1-2\sum_{i=1}^{n}\frac{y_{i}\hat{y_{i}}+\varepsilon }{y_{i}^{2}+\hat{y_{i}^{2}}+\varepsilon })$$

(10) $$bce_{{jaccard}_{loss}}=\left(-\frac{1}{N}\sum_{i=1}^{n}y_{i}\cdot log\hat{y_{i}}+(1-y_{i})\cdot log(1-\hat{y_{i}})\right)+\;\left(1-\frac{TP}{TP+FP+TN}\right)$$

(11) $$bc{e\_focal}_{{dice}_{loss}}=\left(\sum_{i=0}^{n}\alpha_{t}\cdot {\left(1-\hat{y_{i}}\right)}^{\gamma }\cdot log(\hat{y_{i}})\right)+\;(1-2\sum_{i=1}^{n}\frac{y_{i}\hat{y_{i}}+\varepsilon }{y_{i}^{2}+\hat{y_{i}^{2}}+\varepsilon }),\left\{\begin{array}{c} \alpha =0.25\\ \gamma =2 \end{array}\right.$$

(12) $$bc{e\_focal}_{{jaccard}_{loss}}=\left(-\frac{1}{N}\sum_{i=1}^{n}y_{i}\cdot log\hat{y_{i}}+(1-y_{i})\cdot log(1-\hat{y_{i}})\right)+\;\left(1-\frac{TP}{TP+FP+TN}\right)$$

## 4. Discussion

In this study, two CNN models, Inception-ResNetV2 and U-Net, were utilized for training the network. The core concept of Inception-ResNetV2 is to combine inception modules and residual direct connections using residual connection shortcuts to successfully train deeper neural networks, while significantly simplifying inception modules. As indicated in Figure 15, the structure of Inception-ResNetV2 is divided into several parts: Stem, Inception-resnet-A, Reduction-A, Inception-resnet-B, Reduction-B, and Inception-resnet-C. By combining these modules, Inception-ResNetV2 can achieve a deep network architecture without encountering the problems of gradient disappearance and can converge better. Recently, Inception-ResNetV2 has been increasingly used for medical image recognition. For example, a previous study [57] explored the use of Inception-ResNetV2 for brain tumor detection. Other studies have also applied Inception-ResNetV2 in various applications, such as skin lesion classification methods [58,59] and benchmark testing for aortic pathology analysis [60].

Proposed in 2015, U-Net is widely used in medical image segmentation owing to its unique structure [61], as shown in Figure 16. Its structure can be considered as an encoder–decoder structure. The encoder consists of four sub-modules, each containing two convolutional layers, followed by a max pooling layer for down-sampling, which gradually decrease the resolution of the image. The decoder consists of four submodules that gradually increase the resolution of the image by up-sampling until it is consistent with the input image resolution. Several improved versions based on U-Net are available, such as 3D U-Net [62], which is used for the segmentation of 3D images; Res-UNet [63], which combines the concept of ResNet using residual blocks instead of convolutional layers; and ResUnet++ [64], which introduces attention modules [65] and ASPP modules [66].

In this study, KUB images were used to train the model. According to a systematic review of the latest advancements in the use of artificial intelligence in urology conducted by Dai et al. [70], only one study used KUB images [30]. However, recent research [22] has demonstrated that image preprocessing techniques coupled with model classification could enhance the accuracy of renal stone detection. In this aspect, our results surpassed those of ref. [22]. Other studies, such as that by Parakh et al. [71], had primarily considered machine learning and DL models based on CT images. The advantages of plain film X-ray images include their low dosage and cost, which enable their use across a wide range of medical institutions. However, DL models struggle to accurately detect small objects or features, and renal stones in a KUB image typically occupy only a small number of pixels [72]. To resolve this issue, we cropped the images to magnify the renal stones, thereby facilitating model training.

First, we classified the KUB images based on the presence or absence of renal stones and masked the images with renal stones after classification. The preprocessed and classified renal stone images have reduced misjudgments during segmentation. The segmented stone positions will further assist physicians in diagnosis. Our CAD system has demonstrated that X-ray images can be effective in detecting renal stones, offering a promising research direction and providing an alternative system in renal stone diagnosis using KUB imaging, in addition to CT imaging. While research on the use of plain film X-ray images to detect renal stones is sparse, the results of this study are promising and indicate bright prospects for future research.

## 5. Conclusions

In this study, we proposed a computer-aided diagnostic system, which was divided into two subsystems. Both subsystems used CNN models to train the DL models. Subsystem 1 classifies and subsystem 2 segments the urinary stones on KUB images. First, subsystem 1 adopts the image preprocessing procedure designed in this study, for which we proposed a method based on subsystem 1 for image cropping. Images of the entire renal stone can be obtained to the greatest extent possible using a sliding window combined with the classification model, avoiding the division of stones into multiple images for recognition owing to average cropping. Experimental data showed that preprocessing, which included image masking, contrast-limited AHE, and image cropping, helped the model to effectively classify the stones and non-stones. Moreover, the Inception-ResNetV2 model was validated to further improve its accuracy over the ResNet50 model. Based on the experimental data, U-Net can accurately generate a urinary stone mask; however, the MIoU data showed that the accuracy of the background was low, and a few erroneous masks were misidentified as urinary stones. In clinical medicine, conventional radiography for detecting stones may exhibit unique characteristics that are less frequently encountered. Due to the scarcity of such images, it is difficult to train the model effectively. Therefore, most training images used in this study were of stones that could be observed with the naked eye. In the future, if several difficult-to-judge KUB images can be collected and trained using the proposed image preprocessing architecture, the generalization ability of the model can be further improved. This will render the diagnostic tool more reliable and enhance its potential. In this study, although the U-Net model in subsystem 2 achieved good performance, misjudgment of feature masks is a problem that needs to be addressed in future research. Some ribs, gas, or fecal matter commonly present in KUB images can cause dense white areas in the images, which are uncontrollable factors that cannot be removed by image masking, such as in the spine and pelvis. Solving these problems is a direction for future research.

## Figures and Tables

**Table 1:** Hardware information.

CPU	Graphics Card	Memory
Intel Core i7-8700 @ 3.19 GHz	Nvidia GeForce RTX3070 8 G	32 G

**Table 2:** Model initialization parameters.

Epochs	50
Batch size	16
Learning rate	0.001
Loss function	Binary cross-entropy
Optimization algorithm	Ranger

**Table 3:** Confusion matrix of the test set for the ResNet50 classification model.

		Predicted Label	
		Have Stone	NO Stone
True label	Have stone	182	3
	No stone	1	184

**Table 4:** Evaluation metrics performance of ResNet50 classification model on the test set.

	Accuracy	Sensitivity	Specificity	Precision	F1-Score
Testing dataset	0.989	0.995	0.984	0.984	0.989

**Table 5:** Confusion matrix of the test set for the Inception-ResNetV2 classification model.

		Predicted Label	
		Have Stone	No Stone
True label	Have stone	184	0
	No stone	1	185

**Table 6:** Evaluation metrics performance of Inception-ResNetV2 classification model on the test set.

	Accuracy	Sensitivity	Specificity	Precision	F1-Score
Testing dataset	0.997	1.000	0.995	0.995	0.997

**Table 7:** ResNet50 and Inception-ResNetV2 evaluation results comparison.

	ResNet50 [31]	Inception-ResNetV2
Accuracy	0.989	0.997
Sensitivity	0.995	1.000
Specificity	0.984	0.995
Precision	0.984	0.995
F1-score	0.989	0.997

**Table 8:** U-net model initialization parameters.

Epochs	100
Batch size	8
Learning rate	0.0001
Loss function	Focal loss + Jaccard loss
Optimization algorithm	Ranger

**Table 9:** Confusion matrix of ResNet34 using Bce_dice_loss, Bce_jaccard_loss, binary_focal_dice_loss, and binary_focal_jaccard_loss.

	Bce_dice_loss	Bce_jaccard_loss	Binary_focal_dice_loss	Binary_focal_jaccard_loss
TP	270,382	267,678	259,717	268,203
FP	72,813	75,517	83,478	74,992
TN	1,480,475	1,480,115	1,481,879	1,482,633
FN	26,330	26,690	24,926	27,751

**Table 10:** Confusion matrix of ResNet50 using Bce_dice_loss, Bce_jaccard_loss, binary_focal_dice_loss, and binary_focal_jaccard_loss.

	Bce_dice_loss	Bce_jaccard_loss	Binary_focal_dice_loss	Binary_focal_jaccard_loss
TP	268,540	270,597	256,915	266,816
FP	74,655	72,598	86,280	76,379
TN	1,479,054	1,472,688	1,487,191	1,480,639
FN	27,751	34,117	19,614	26,166

**Table 11:** Evaluation metrics calculated based on the confusion matrix for ResNet34.

	Bce_dice_loss		Bce_jaccard_loss		Binary_focal_dice_loss		Binary_focal_jaccard_loss	
	Positive	Negative	Positive	Negative	Positive	Negative	Positive	Negative
Accuracy	**0.946**	**0.946**	0.945	0.945	0.941	0.941	**0.946**	**0.946**
Sensitivity	**0.953**	0.911	0.951	0.909	0.947	0.912	0.952	0.917
Precision	0.983	0.788	0.982	0.780	0.983	0.757	**0.984**	0.781
F1-score	**0.968**	0.845	0.967	0.840	0.965	0.827	**0.968**	0.844
IoU	**0.937**	0.732	0.935	0.724	0.932	0.706	**0.937**	0.730
MIoU	**0.834**	**0.834**	0.830	0.830	0.819	0.819	**0.834**	**0.834**
FWIoU	0.904	0.904	0.902	0.902	0.897	0.897	**0.905**	**0.905**

**Table 12:** Evaluation metrics calculated based on the confusion matrix for ResNet50.

	Bce_dice_loss		Bce_jaccard_loss		Binary_focal_dice_loss		Binary_focal_jaccard_loss	
	Positive	Negative	Positive	Negative	Positive	Negative	Negative	Positive
Accuracy	**0.945**	**0.945**	0.942	0.942	0.943	0.943	**0.945**	**0.945**
Sensitivity	0.952	0.906	**0.953**	0.888	0.945	0.929	0.951	0.911
Precision	0.982	0.782	0.977	0.788	**0.987**	0.749	0.983	0.777
F1-score	**0.967**	0.840	0.965	0.836	0.966	0.829	**0.967**	0.839
IoU	**0.935**	0.724	0.932	0.717	0.934	0.708	**0.935**	0.722
MIoU	**0.830**	**0.830**	0.825	0.825	0.821	0.821	0.829	0.829
FWIoU	0.901	0.901	0.897	0.897	0.900	0.900	**0.902**	**0.902**

**Table 13:** Comparison of comprehensive evaluation indicators between ResNet34 and ResNet50.

	ResNet34′s Bce_dice_loss	ResNet34′s Binary_focal_jaccard_loss	ResNet50′s Bce_dice_loss	ResNet50′s Binary_focal_jaccard_loss
Accuracy	**0.946**	**0.946**	0.945	0.945
Sensitivity	**0.953**	0.952	0.952	0.951
Precision	0.983	**0.984**	0.982	0.983
F1-score	**0.968**	**0.968**	0.967	0.967
IoU	**0.937**	**0.937**	0.935	0.935
MIoU	**0.834**	**0.834**	0.830	0.829
FWIoU	0.904	**0.905**	0.901	0.902

## Data Availability

The data presented in this study are available on request from the corresponding author.
